# Stopping circulatory vaccine-derived poliovirus in Kaduna state by scaling up special interventions in local government areas along rivers of interest- kamacha basin experience, 2013–2015

**DOI:** 10.1186/s12889-018-6180-4

**Published:** 2018-12-13

**Authors:** Audu I. Musa, Faisal Shuaib, Fiona Braka, Pascal Mkanda, Richard Banda, Charles Korir, Sisay G. Tegegne, Suleiman Abdullahi, Gregory C. Umeh, Terna I. Nomhwange, Hadiza Aliyu Iyal, Sambo Ishaku, Usman Adamu, Eunice Damisa, Murtala Bagana, Victor Gugong, Hadiza Balarabe, Peter Nsubuga, Rui G. Vaz

**Affiliations:** 1 World Health Organization, Abuja, Nigeria; 2 National Primary Health Care Agency, Abuja, Nigeria; 3 Emergency Operations Centre (sEOC), Kaduna, Nigeria; 4 Kaduna State Primary Health Care Agency, Kaduna, Nigeria; 5 Global Public Health Solutions, Atlanta, GA USA

**Keywords:** Circulatory vaccine derived polio-virus, Special interventions, Nigeria

## Abstract

**Background:**

The Kamacha river is one of the five polio environmental surveillance sites in Kaduna State where 13 circulating vaccine-derived polioviruses (cVDPDs) were isolated between 2014 and 2015. Kamacha river accounted for 5 of all reported cVDPVs in Kaduna State between 2014 and 2015. Poor quality Supplemental Immunization Activities (SIAs) and low population immunity have been reported in the 10 LGAs with tributaries that flow into the river. We described the processes of implementing the various health interventions in these targeted LGAs along the Kamacha River and assessed the effectiveness of the interventions in stopping cVDPV in Kaduna, state, Nigeria.

**Methods:**

Special interventions that had been proven to be functional and effective in reaching unreached children with potent vaccines in the state were scaled up in these targeted 10 LGAs along the Kamacha River. These interventions included revision of house based microplans, scaling up of transit vaccination, scaling up of youth engagement, intensified supportive supervision, scaling up of Directly Observed Polio Vaccination (DOPV) and in-between rounds vaccination activities. We analyzed immunization plus days (IPDs) administrative tally sheet and monitoring data from 10 rounds before and 10 rounds after the special interventions.

**Results:**

The number of children immunized increased from 1,862,958 in December 2014 before the intervention to 1,922,940 in March 2016 after the intervention.

Lot Quality Assurance Sampling (LQAS) results showed an increase in the proportion of LGAs accepted at coverage > 90% after the interventions, from 67% before intervention to 84% after intervention. The proportion of non-polio AFP children with > 4 doses of oral polio vaccine increased from 2 to 8% before to 93–98% after the interventions.. No new environmental cVDPV has been isolated since the introduction of the interventions in April 2015 until July 2016.

**Conclusion:**

Scaling up known working interventions in the 10 LGAs with tributaries that drain to Kamacha River environmental sample site may have contributed to improved immunity and interruption of cVDPV in Kaduna state. These interventions should be replicated in LGAs and states with persistent poliovirus isolation.

## Background

As progress towards wild poliovirus eradication accelerated in the late 1990s, new risks to a polio-free world became apparent. Vaccine-derived polioviruses (VDPVs) can both circulate and paralyze, causing polio outbreaks due to circulating VDPVs (cVDPVs).Immune vaccine-derived polioviruses (iVDPVs) may cause paralysis in some individuals with primary immunodeficiency [1–5]. In May 2008, in line with guidance from the World Health Organization (WHO)‘s Scientific Advisory Group of Experts on immunization (SAGE), the World Health Assembly (WHA) endorsed the principle of synchronized oral polio vaccine (OPV) cessation globally, to reduce the incidence of cVDPV [6].

To strengthen surveillance for polioviruses, the WHO recommends complementary surveillance by introducing environmental surveillance [7, 8]. As fewer wild poliovirus (WPVs) are detected, the role of environmental sampling will increase; in addition to its use for detection of potential VDPVs. Nigeria introduced environmental surveillance in 2011 in Kano State, and it was expanded to Kaduna State in 2013 starting with three sites of Rigassa River in Igabi, Limanchi Kona Bridge, and Kamacha River in Sabon Gari, and Zaria local government areas (LGAs). The two other sites of Kusfa Bridge and Ungwan Jaba of Zaria and Sabon Gari LGAs were added in 2015 to increase sensitivity. Despite several rounds of polio supplemental immunization activities (SIAs), there remained sanctuaries with the persistent transmission of cVDPV in Kaduna State [4, 8, 9]. A total of five cVDPV2 from AFP were isolated in 2013, 30 in 2014 and one in 2015 (10). Nigeria contributed 41% of cVDPVs in Africa between 2012 and 2016 and 23% of the cases in Nigeria were from Kaduna State [10]. A total of 13 cVDPVs were isolated from the environment in 2014 and 2015 of which 11 (84.6%) were from Kamacha River site in Zaria LGA and the remaining 15.4% from Limanchi Kona Bridge site in Zaria and Rigassa River site in Igabi LGA [11, 12].

Supplemntal Immunization Activities, monitoring data and supervision report showed poor quality SIAs and potential low population immunity in the LGAs along this river. The root causes ranged from persistent poor team performance by vaccination team members, refusal of immunization by caregivers, and poor micro-planning, and a high number of unimmunized children from poor routine immunization coverage [13–16].

To address low population immunity and poor vaccination team performance, special interventions that were proven to be functional and effective in the state were scaled up in these targeted 10 LGAs along the Kamacha River. These interventions included revision of household based micro plans (involved listing of all major and minor settlements and enumeration of all under-5 years old children), scaling up of transit vaccination (for examples motor parks, check points, markets vaccinations), scaling up of youth engagement as well as intensified supportive supervision (youth accompanied vaccination teams working in volatile or security compromised settlements). Others were scaling up of Directly Observed Polio Vaccination (DOPV) (immunization outside the households two to 3 days before the vaccination teams commence house-to-house vaccination) and in-between rounds vaccination activities (vaccination immediately after a campaign targeted at under-performing settlements) [17–19].

We described the processes of implementing the various health interventions in the 10 targeted LGAs along the Kamacha River and assessed the effectiveness of the interventions in stopping cVDPV, Kaduna state, Nigeria.

## Methods

### Targeted area

**Fig. 1:**
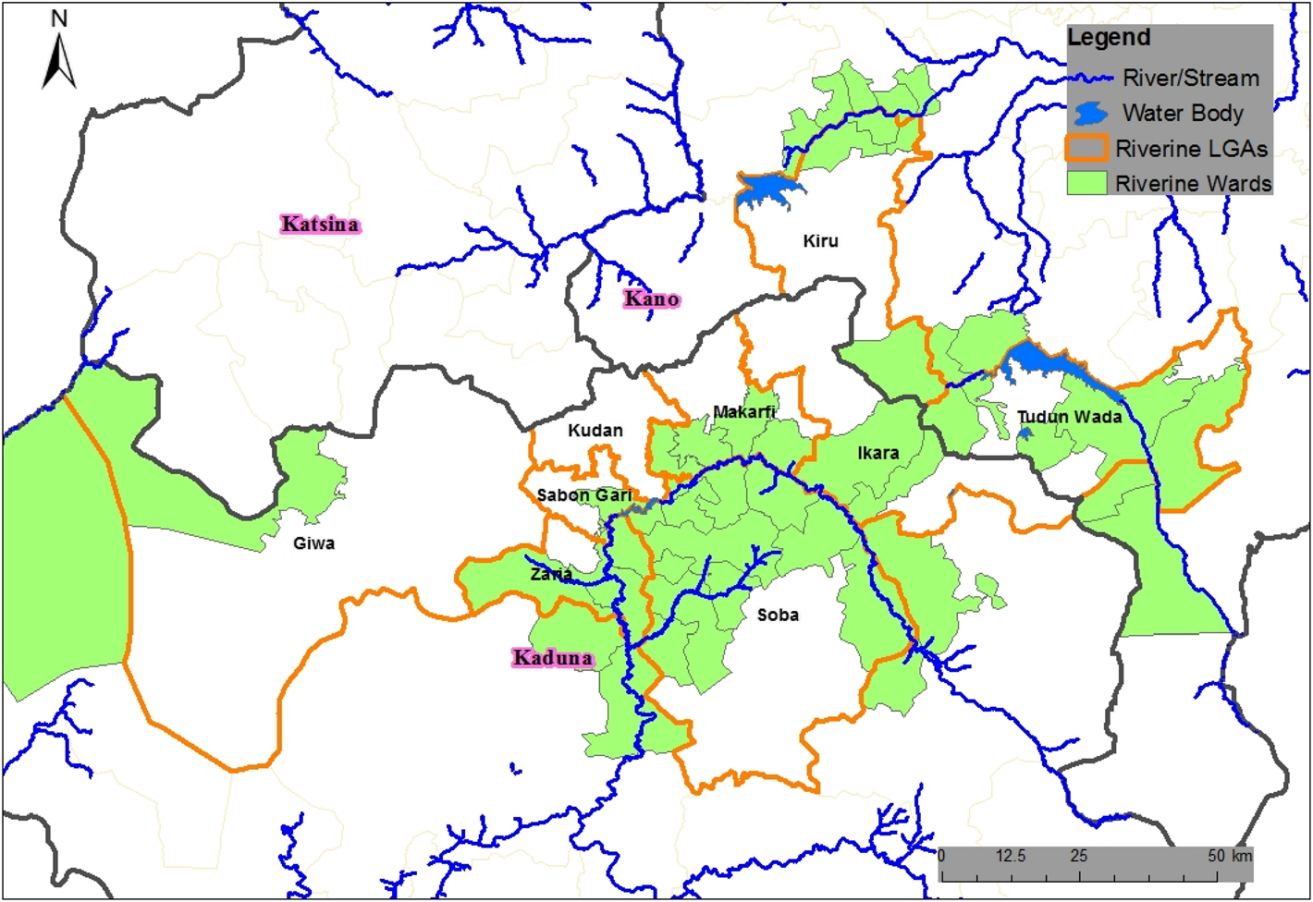
Map showing tributaries from 10 LGAs in Kaduna state that drain into to river Kamacha, April 2015

We targeted the 10 LGAs with tributaries to Kamacha River in Zaria LGA. The 10 LGAs were Zaria, Sabon Gari, Kudan, Giwa, Soba, Kubau, Makarfi, Ikara, Birnin Gwari and Igabi. Tributaries of rivers from 10 LGAs in Kaduna State drained into Kamacha River in Zaria (Fig. 1). Tributaries from Sabuwa and Danja LGAs of Katsina passed through Birnin Gwari, Igabi and Kudan LGA to drain to Kamacha while another tributary from Makarfi LGA passed through Kudan, Giwa and Sabon Gari LGAs to drain into the Kamacha River. The last route was from Bauchi state through Kubau, Ikara and Soba LGAs to the Kamacha River.

### Study design

We implemented six special interventions in the 10 LGAs in addition to the routine activities that were being conducted during SIAs (Table 1). We implemented the special interventions in phases from pre-campaign, intra-campaign, and post-campaign for each polio SIA. We evaluated the impact of the interventions by assessing the proportion of LGAs with LQAS accepted at coverage ≥90% by polio vaccination activities before and after the special interventions in the 10 LGAs with tributaries to Kamacha River.

**Table 1:** Description of special interventions conducted in the 10 local government areas with tributaries to the Kamacha River- Kaduna, Nigeria, 2014–2016

Intervention	Description	Where it was used before in Nigeria	When we started using it in Kamacha
1) Walk-through Micro plans	Enumeration and line-listing of households and children < 5 years and < 1 year of age in each them.	Kano state in September 2013	April 2015
2) DOPV	Directly observed outside OPV vaccination of children. It was used exclusively for the first 2–3 days of each round of OPV vaccination	Bauchi and Kano, in year in August 2014	Introduced to Kamacha LGAs in September 2014 and scaled up in April 2015
3) Youth engagement	Youth engaged in wards and settlements with high resistant of polio vaccination and Vaccination team harassment	Rigassa in Igabi LGA Kaduna state from May 2014	Introduced to Kamacha LGAs in September 2014 and scaled up in April 2015
4) Transit points vaccination	Vaccination in motor parks, roads blocks and markets	Borno, Yobe, and Taraba	Introduced to Kamacha LGAs in September 2014 and scaled up in April 2015
5) In-between rounds Vaccination	Vaccination in settlements with high missed children during SIAs or settlements with potential immunity gaps.	Borno, Yobe in 2013	April 2015
6) Engagement of Independent monitors	Monitor implementation of planned activities (in-process)	Kaduna & other states	April 2015

### Interventions

We conducted the following interventions: household based micro plans (involved listing of all major and minor settlements and enumeration of all < 5 years old children), scaling up of transit vaccination (for examples motor parks, check points, markets vaccinations), scaling up of youth engagement as well as intensified supportive supervision (youth accompanied vaccination teams working in volatile or security compromised settlements). Others were scaling up of Directly Observed Polio Vaccination (DOPV) (immunization outside the households two to 3 days before the vaccination teams commence house-to-house vaccination) and in-between rounds vaccination activities (vaccination immediately after a campaign targeted at under-performing settlements) (Table 1 & 2).

**Table 2:** Special interventions introduced in the 10 local government areas with tributaries to the Kamacha River- Kaduna, Nigeria, 2014–2016

Intervention	Before Scaling UP	After Scaling UP
No of Independent Monitors (in-process)	40	96
No Youth Engagement	120	338
No of Transit Points for Vaccination	4	32
Revised Household Based Microplan (no of LGAs)	0	3
DOPV Activities		
No of DOPV Days	2	3
No of DOPV Teams	1270	2407
No of DOPV Supervisors	423	2294
In Between Round Activities with New Attractive Pluses Added (Nodules)	0	1

### Pre-campaign

The major special intervention implemented at this phase was improved micro planning. We conducted community participative physical walk-through and micro-census in the catchment areas and settlements 1000 m from the course of the rivers. The process involved enumeration of the total number of households and eligible children < 5 and < 1 year of age in all the households in the catchment settlements. The plans were used for SIAs, routine immunization and in-between rounds vaccination activities.

### Intra-campaign

Special interventions implemented at this phase included expanded Directly Observed Polio Vaccination (DOPV), youth engagement for vaccination, transit points’ vaccination, and in-between round activities.

Furthermore, we introduced intra-campaign mock lot quality assurance sampling (LQAS) surveys in these priority LGAs conducted on day 3 and day 4 of polio SIAs to assess the coverage in wards and settlements already completed during the exercise. The trained LQAS surveyors were deployed to sample 60 households per day per LGA for the 2 days. The results were presented at the daily evening review meetings to the LGA team to initiate immediate plans for revisits or take other necessary actions to vaccinate the missed children and correct poor performing teams.

### Post-campaign and in-between rounds

At the end of each round of vaccination, we prioritized settlements with a record of a high number of missed children or poor access during the SIA for in-between rounds activities. The State Emergency Operations Center (EOC) organized a 1-day feedback session with the various stakeholders to discuss issues and challenges during the vaccination rounds. The wards and settlements with a high proportion of missed children or with potential immunity gaps were targeted for in-between rounds vaccination using the various strategies mentioned before.

### Primary outcomes

The primary outcomes were children immunized by the transit and DOPV teams during SIAs and by the in-between round teams after SIAs; number of cVDPV isolated after the interventions and the number of OPV doses received by children with non-polio associated paralysis.

### Data collection

We collected data from the vaccination teams’ tally sheets, weekly surveillance, laboratory results on OPV doses of non-polio associated paralysis, and cVDPV isolation and Lots Quality Assurance Sampling (LQAS).

### Data analysis

We analyzed the number of children immunized by polio vaccination teams during SIAs and in-between rounds activities; trend in the number of oral polio vaccine doses received by children with non-polio associated acute flaccid paralysis; and trend of cVDPV before and after the special interventions in the 10 LGAs with rivers that drained to Kamacha river.

We also analyzed the proportion of LGAs with LQAS accepted at coverage ≥90% by polio vaccination activities before and after the special interventions in the 10 LGAs with tributaries to Kamacha River. We used IPDs data from March, April, May, June, August, September, November to December of 2014, and January and March of 2015 compared with April, June, July, August, September, October to December of 2015, and January, February, and March of 2016.

## Results

Data from tally sheets showed more children vaccinated in each round of polio vaccination after the intervention. There was an increase from the highest immunized before the intervention of 1,862,958 in December 2014 to the highest immunized after the intervention of 1,922,940 in March 2016 (Table 3 & 4).

**Table 3:** Children immunized by special interventions during in-between rounds activities in the 10 local government areas with tributaries to the Kamacha River- Kaduna, Nigeria, 2014–2016

Intervention	Qtr2 2015	Qtr3 2015	Qtr4 2015	Qtr1 2016	Qtr2 2016	Sub Total
Market Vaccination	85,194	93,318	108,270	254,633	4,311,815	973,230
Motor Park Vaccination	123,604	122,560	135,093	149,988	49,967	580,212
FRSC Check Point Vaccination	12,110	19,115	143,293	24,121	11,413	81,052
Youth DOPV vaccination	73,894	57,443	79,791	193,564	405,648	810,340
Permanent Hospital vaccination	28,217	28,670	40,030	121,087	149,737	367,741
Cross Border Vaccination	14,845	17,608	14,166	21,180	13,340	81,139
Nomadic Route vaccination			9558	18,339	36,723	64,620
Hit and Run vaccination					17,487	17,487
Total	336,864	338,714	401,201	782,912	1,116,130	2,975,821

**Table 4:** Number of children immunized by Polio vaccination activities (tally sheet data) before and after the special interventions in the 10 local government areas with tributaries to the Kamacha River - Kaduna, Nigeria, 2014–2016

IPDs Round	Before Intervention	After Intervention
Round 1	1,703,166	1,841,964
Round 2	1,656,280	1,872,952
Round 3	1,720,446	1,839,950
Round 4	1,715,668	1,880,567
Round 5	1,743,582	1,754,820
Round 6	1,782,709	1,764,809
Round 7	1,840,821	1,902,968
Round 8	1,862,958	1,906,804
Round 9	1,881,866	1,916,276
Round 10	1,829,370	1,922,940

Lots Quality Assurance Sampling results showed an increase in the proportion of LGAs accepted at coverage > 90% after the intervention. The proportion of the LGAs accepted at coverage > 90% was higher in 7 of the 10 IPDs rounds after the intervention while the proportion of the LGAs accepted at coverage > 90% was higher in three IPDs rounds before the intervention (Table 5).

**Table 5:** Proportion of LGAs with LQAs accepted at coverage ≥90% by Polio vaccination activities before and after the special interventions in the 10 Local Government Areas with tributaries to Kamacha River- Kaduna, Nigeria, 2014–2016

IPDs Round	Before Intervention (%)	After Intervention (%)
Round 1	18	61
Round 2	35	56
Round 3	50	78
Round 4	76	78
Round 5	94	71
Round 6	80	71
Round 7	71	61
Round 8	72	80
Round 9	67	90
Round 10	67	84

Ninety percent of the children with non-polio associated acute flaccid paralysis had four or more OPV doses after the intervention (Table 6).

**Table 6:** Trend in the number of Oral Polio Vaccine Doses Received by children with Non-Polio Associated Acute Flaccid Paralysis (NPAFP) in the 10 Local Government Areas with tributaries to Kamacha River- Kaduna, Nigeria, 2014–2016

LGA	2013			2014			2015			2016		
	0 Doses (%)	1–3 Doses (%)	> 4 Doses (%)	0 Doses (%)	1–3 Doses (%)	> 4 Doses (%)	0 Doses (%)	1–3 Doses (%)	> 4 Doses (%)	0 Doses (%)	1–3 Doses (%)	> 4 Doses (%)
Birnin Gwari	10	0	90	0	0	100	0	8	92	0	8	92
Giwa	0	8	92	0	7	93	0	5	93	0	0	100
Igabi	0	32	68	0	10	90	0	8	92	0	5	95
Ikara	0	0	100	0	5	95	0	0	100	0	0	100
Kubau	0	0	100	0	0	100	0	0	100	0	0	100
Kudan	0	11	89	9	0	91	0	0	100	0	0	100
Makarfi	0	14	86	0	0	100	0	0	100	0	0	100
Sabon/ Gari	7	20	73	0	22	78	0	0	100	0	4	96
Soba	0	0	100	6	0	94	0	6	94	0	0	100
Zaria	9	9	82	0	38	63	0	12	88	0		

**Fig. 2:**
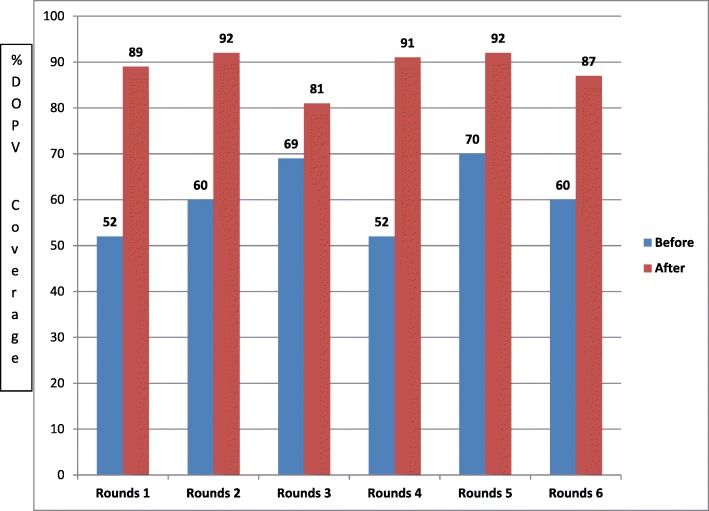
Number of children immunized from DOPV intervention during IPDs rounds in the 10 Local government areas with rivers that drained to Kamacha river- Kaduna, Nigeria, 2015–2016

There was an increase in the number of vaccinated children (by tally sheet) from DOPV in all the IPDs rounds after scaling up the intervention (Fig. 2). In most of the rounds > 80% of the target children were vaccinated through DOPV, the highest were in rounds two and five (June and September 2015). The highest contribution of DOPV before the scaling up was 70% in the month of January 2015.

**Fig. 3:**
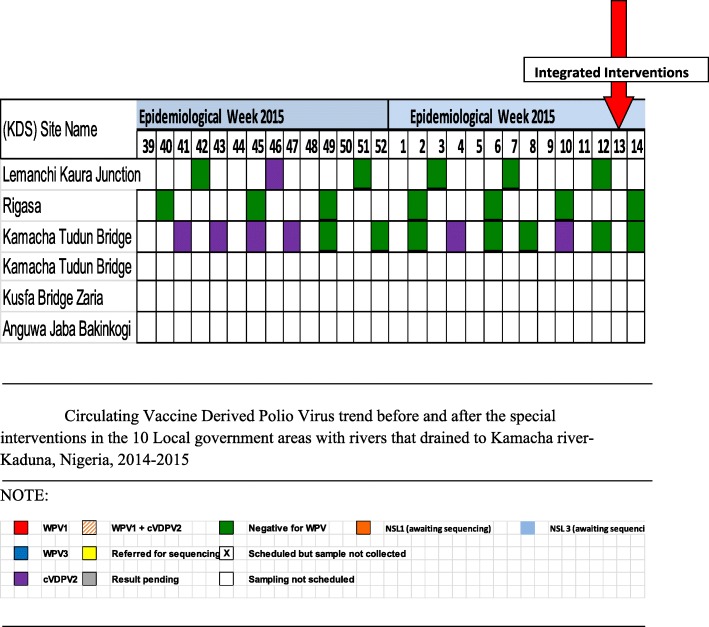
Circulating Vaccine Derived Polio Virus trend before and after the special interventions in the 10 Local government areas with rivers that drained to Kamacha river- Kaduna, Nigeria, 2014–2015

The trend of environmental sample results from Nigeria weekly polio statistics showed no new environmental cVDPV isolated after the introduction of the interventions in April 2015 (Fig. 3). The last virus isolated in the state was in week 10 of 2015.

## Discussion

We found that scaling up the six interventions that were known to work in the 10 LGAs with tributaries that drain to Kamacha River environmental sample site, may have contributed to improved immunity and interruption of cVDPV in the state. Human and financial resources were targeted to the areas with known gaps rather than generalizing the utilization of the resources with little effect.

We also found that introduction of the multiple working interventions in settlements with noncompliance improved the quality of household based micro plans and intra-campaign monitoring. These interventions resulted in reaching more children during polio SIAs with a reduction in missed children due to noncompliance and child absence [20, 21].

Furthermore, we found that scaling up of validated vaccination, just as in tuberculosis treatment using directly observed treatment short course (DOTS) accelerated the process of improving population immunity in the noncompliance community. It ensured vaccination team members do not connive with caregivers from non-compliant households to finger mark the children without actually vaccinating them with OPV during polio SIAs [19].

The study also revealed the value of vaccinating children in special places as nomadic routes and security compromised settlements. Despite the low number of children vaccinated in these special areas, they are highly valued children, who are sometimes missed during polio SIAs [22]. We also found that sustaining some workable interventions beyond polio SIA days (by implementing in-between rounds vaccination) contributed to bridging immunity gaps in the vulnerable communities with records of persistently missed children.

## Limitations

Other interventions were concurrently being implemented during the study period. Some of the improved outcomes demonstrated by this study may be equally attributable to them.

## Conclusion

Scaling up of working innovations in communities with a record of immunity gaps is essential to improving the quality of SIA and interruption of polioviruses in a shorter period hence reducing the long-term cost of additional SIAs.

## Recommendations

We recommend similar interventions in riverine communities with persistent poliovirus transmission. We recommend studies on the cost effectiveness of the scaled-up interventions. These studies should be done in the context of cost per innovations and a potential number of IPDs rounds to be conducted with or without the interventions.

## Abbreviations

cVDPV: Circulatory Vaccine Derived Poliovirus
DOPV: Directly Observed Polio vaccination
DOTS: Directly Observed Short Course
EOC: Emergency Operations Centre
IPDs: Immunization Plus Days
iVDPV: Immunodeficiency Vaccine Derived Poliovirus
LGA: Local Government Area
LQAS: Lots Quality Assurance Sampling
OPV: Oral Polio Vaccine
SAGE: Scientific Advisory Group of Experts
SIAs: Supplemental Immunization Days
VDPV: Vaccine Derived Poliovirus
WHA: World Health Assembly
WHO: World Health Organization
